# Two Successful Cataract Operations on a Dog

**Published:** 1895-03

**Authors:** Robert L. Randolph


### THE JOURNAL
OF
### COMPARATIVE MEDICINE AND VETERINARY ARCHIVES.

VOL. XVI. *MARCH*, 1895. No. 3.

### TWO SUCCESSFUL CATARACT OPERATIONS ON A DOG.

BY ROBERT L. RANDOLPH, M.D.

It is generally known that cataract is not uncommon in the lower animals and especially in the dog and horse. In the horse cataract is apt to be the result of recurrent irido-choroiditis, and this latter affection as seen in the horse is remarkable for its tendency to relapses, appearing often periodically and representing the disease known as "moon-blindness." Cataract, then, in the horse is almost always inflammatory in its origin.

When cataract is found in the dog there is no such history of a coincident inflammation, but we find a condition that differs little from the same affection in man. With regard to the operation for cataract in the dog no special difficulties are presented as contrasted with the same operation in man, except that we are compelled to use a general anæsthetic in the former case, and this is a disadvantage. Cocain has undoubtedly lessened the gravity of cataract operations.

It is evident that the healing process in animals is surrounded with far greater dangers than is the case with human beings, and it is this no doubt that deters us from operating for cataract in the lower animals.

In the first part of October, 1894, A. W. Clement, V. S., of this city, brought to my office a handsome pointer dog. The dog was perfectly blind, and had been sent to Dr. Clement for relief. He was eighteen months old and in fine physical condition generally. His master said that he had been going blind for three months, and at the end of that time only light perception was left. On the street he would crouch at his master's feet at the sound of an approaching vehicle, and could not be dragged away till the vehicle had passed. When brought to my office and allowed to smell around the room he ran into the wall and chairs at almost every turn. In being led one had to pull him along, as he was fearful of running into objects. On examination I found both eyes free from irritation. The pupils quickly responded to light. With the ophthalmoscope it could readily be seen that the lenses were opaque, and on using a mydriatic I found that they were uniformly and entirely opaque. The color presented by the cataract was more a milk-white than gray, not unlike what we see in the ordinary traumatic cataract when there has been extensive laceration of the anterior capsule, and the whole lens has become immediately opaque. A similar appearance is presented by the so-called naphthalin cataract that I have produced in rabbits by feeding them on naphthalin in the manner described by Dor, Panas, and others. Such cataracts belong to the variety known as soft cataract. I determined to perform discission, so the dog was first given a hypodermatic injection of morphia, and then chloroformed. My instruments were boiled a half-hour before using them. Only two instruments are necessary, the needle and fixation forceps. The lids were held open by an assistant. At this time I operated on the right eye, and I may add that the pupil of the eye had been well dilated with atropia before the operation. The needle was passed into the cornea in the usual way, and a crucial incision was made in the anterior capsule. Atropia was instilled, and the dog was put into a small kennel and allowed to recover from the effects of the chloroform. The next day there was a large mass of cortical substance protruding into the anterior chamber. The recovery was absolutely uneventful, and at no time were there symptoms of irritation. At the end of the first week Dr. Clement observed that the dog went about the stable-yard with greater freedom. He was confined to his kennel for two weeks, and whenever left out into the yard he was closely watched by the stable-man. In three weeks there was a perfectly clear pupil, and only within the extreme ciliary margin of the pupil were there any remains of the capsule to be seen. During this time atropia was dropped in the eye three times a day. At the end of three weeks I made the following test: I arranged several chairs in such a manner as to form a zig-zag path leading from one room through a narrow door into the adjoining room, and then went into the adjoining room and called to the dog. He came along the path laid out for him without a pause. This he did several times without striking a chair. I then placed a chair in the doorway and called to him, and he jumped over the chair to me without the slightest hesitation. That day I operated on the other eye and used cocain. The dog was exceedingly restless, and had to be held down. This restlessness was not due to pain but to nervousness, but it was enough to convince me that a general anæsthetic is indispensable. The operation was similar to the first one, and in five weeks there was to be seen only a small band of capsule lying in the pupillary area.

At this time it was impossible to detect anything wrong with the dog's vision. He moved about with freedom and rapidity, and ten days later his master, Mr. W. T. Wilson, of this city, wrote me that he had taken the dog out on a hunt, and had found him just as efficient as ever in so far as his hunting qualities were concerned, and that he jumped fences and ditches as readily as the other dogs in the field.

It seems, then, that the effect of the operation was to give the dog a vision that is practically perfect. At this time both Dr. Clement and I thought the case a unique one, and as far as I can learn it is unique in this country, but since then I have found quite a number of operations of a similar character reported in foreign journals. Among others, White Cooper,[1] in 1850, gives an interesting account of some successful operations for cataract performed on bears in the London Zoological Garden. Discission was the operation employed. Brogniez[2] reports a case of successful cataract operation on a horse nine yeare old, and Chégoin[3] operated successfully on an ass twenty-one years old.

As a general thing cataract in the lower animals appears in the earlier years of life, and when it occurs in the horse Crisp[4] thinks the cause to be found in bad light and abundant exhalations of ammonia. Crisp is of the opinion that constitutional affections have little, if anything, to do in the causation of cataract in the lower animals, for usually the animals affected are well nourished and live for years. It will be remembered that the physical condition of my case was perfect so far as could be seen. Haltenhoff[5] reports a case of cataract in a dog associated with diabetes; but, on the other hand, Professor Moeller,[6] of the Veterinary School in Berlin, who has operated a number of times for cataract in dogs and horses, has never found diabetes present, and this has also been the experience of Professor R. Berlin.[7] I failed to test the urine in my case, but the general history of the dog would exclude any such complication; and I may add that in those cases of cataracts in rabbits produced by feeding them on naphthalin frequent examinations of the urine failed to show the least evidence of sugar, though some of the clinical symptoms of these cases suggested diabetes, as, for instance, progressive emaciation, hurried breathing, and an excessive flow of urine.

In speaking of the causes, though, of cataract, in dogs more particularly, it is well to note the fact that accommodative strain, which may be a factor in bringing about cataract in man, can here be excluded; and inasmuch as good vision was obtained in the majority of the cases reported, it is not likely that a disease of the retina or choroid had anything to do with the existence of the cataract. Another interesting fact in connection with this case is the rapidity with which the cataract developed. Within three months after the vision began to fail the dog was blind. I was struck by the shortness of the time required for the absorption of the lenses. Ordinarily, even in very young children, it takes at least two months before absorption is complete, while in those who are older a year or more with several discissions is the rule. In the first eye absorption was practically complete in three weeks, and in the fellow eye nearly all the lens substance had disappeared at the end of five weeks. One would be apt to think that so far as usefulness was concerned the dog would be worthless, but it will be remembered that this was not so.

I am sure that the absolute necessity of artificial help in the shape of glasses for cataract patients is much overrated. There are cases on record (quoted by White Cooper) where after the operation for cataract the patients were compelled, for the sake of experiment, to get along without glasses—in other words, to accommodate their eyes to the new refractive conditions, and after a few months they could get along quite comfortably, though, of course, unable to read. The vision of every animal (man included) is no doubt limited to the needs of the animal. It is not likely then that dogs are possessed of human visual acuteness, and it is evident they do not require such vision.

Certainly few, if any, demands are ever made upon the accommodative apparatus of the dog's eye, so that the loss of the crystalline lens would be attended with comparatively little or no inconvenience, and the same may be said of the horse.

Possibly the good sight in these case is to be accounted for by a reproduction of the lens fibres, and in this connection I may refer to the experiments of Cocteau and Leroy d'Etiolles.[8] removal of the crystalline lens in rabbits, dogs, and cats, that These observers found that in a certain length of time after the another lens was formed. It is a curious fact that in several of these experiments the capsule showed no cicatrice, but was perfectly clear, and contained a lens as voluminous and consistent as the lens that was extracted, and differing in no respect from the latter. Gunn[9] reports a case of traumatic cataract that had occurred in a child, where, after the absorption of the cataract, later on in life new lens fibres were demonstrable. By this time a reproduction of lens fibres may have taken place in my case, but we are not justified in attributing the good vision obtained to such a process, for sight improved at the end of the first week, and the formation of new lens fibres would not likely have begun till the absorption of the old lens was complete.

From investigations made in the London Zoological Gardens it has been found that cataract is common to nearly all of the lower animals, but it is a suggestive fact that cataract is most often seen in the two animals nearest man, the dog and horse. Neither extraction nor couching seems to be proper in operating on the lower animals. The impossibility of keeping the animal quiet, or of surrounding it with any of the usual safeguards, is an objection to extraction. While Moeller has had several successful cases of extraction he states that his best results followed discission. As to couching, the danger of intraocular inflammation makes this operation quite as objectionable here as it is in the case of man. In my opinion discission is the only operation applicable to these cases. A general anæsthetic is indispensable. A bandage should not be applied, as it attracts the dog's attention to his eye, and it will be rubbed or torn off, and injury to the eye would result. It is imperative to use a sterile knife, for we cannot here nullify the effects of infection by careful after-treatment, as we sometimes do in man. A small kennel is necessary, so that no temptation is offered to the dog to move about; and finally a 1 per cent. solution of atropia should be dropped in the eye three times daily during the first three weeks. As regards the anæsthetic to be used, my preference—from considerable experience—is in favor of ether, and I have always been under the impression that it was particularly unsafe to administer chloroform to dogs. Dr. Clement tells me that he always gives chloroform, and precedes the administration of it with a hypodermatic injection of morphia, and that he has never had any unfortunate results, a fact which he thinks is explained by the administration of the morphia.

LITERATURE.

1. An account of operations for cataract on bears. By White Cooper, F. R. C.S. Med. Times, New Series, Vol. I., page 621. London, 1850.
2. Extraction du cristallin chez le cheval, par A. J. Brogniez. Annales d'Oculistique, 1843.
3. Opération de cataracte sur un âne, par Chégoin. Bull. de la Soc. de Chirur. de Paris, 425.
4. Specimens of cataract and of opacities of the cornea in the lower animals. Edwards Crisp, M.D. Trans. Path. Soc. London, Vol. XXII., 350.
5. Klinische Mittheilungen, von G. Haltenhoff. Zeitschr. für vergleich. Augenheilk., 1885, p. 65.
6. Casuistische Mittheilungen über das Vorkommen, und die operative Behandlung des grauen Staares beim Hunde, von Prof. Dr. H. Moeller. Ibid., 1886, pp. 138–146.
7. Beobachtungen über Staar und Staaroperationen bei Thieren, von Prof. Dr. R. Berlin. Ibid., 1887, 59–76.
8. Expériences relatives à la reproduction du cristallin, par MM. Cocteau et Leroy d'Etiolles. Journal de Phys. exper. et pathol., Tom. VII., 30–44.
9. Trans. Oph. Soc. Unit. Kingdom, London, 1888, VII., 126.

---

HAMBURG, Germany, has just quarantined against England and Ireland. Another blow at the American export cattle industry, as through the ports of these countries many of the American cattle are shipped.

THE death of the noted pointer dog "Kent Elgin" from rabies, after treatment at the Pasteur Institute in New York, he having been bitten previously by a common cur supposed to have been rabid, proved a great disappointment among dog fanciers. After treatment he was shipped home, supposedly safe from the possible development of the disease, but two days later developed unmistakable symptoms, and died twenty-four hours later, showing the most intense and violent symptoms.

